# Ecological generalism and physiology mediate fish biogeographic ranges under ocean warming

**DOI:** 10.1098/rspb.2023.2206

**Published:** 2024-01-31

**Authors:** Chloe Hayes, Angus Mitchell, Camille Mellin, David J. Booth, Timothy Ravasi, Ivan Nagelkerken

**Affiliations:** ^1^ Southern Seas Ecology Laboratories, School of Biological Sciences, The University of Adelaide, DX 650418, Adelaide, South Australia, Australia; ^2^ School of the Life Sciences, University of Technology Sydney, Ultimo, New South Wales, Australia; ^3^ Marine Climate Change Unit, Okinawa Institute of Science and Technology Graduate University, 1919-1 Tancha, Onna-son, Okinawa, Japan; ^4^ Australian Research Council Centre of Excellence for Coral Reef Studies, James Cook University, Townsville, Queensland, Australia

**Keywords:** climate change, tropicalization, ecological niche, physiology, coral reef fish, range extending

## Abstract

Climate-driven species redistributions are facilitated by niche modifications that increase a species's chances of establishment in novel communities. It is well understood how range-extending species adjust individual niche traits when entering novel environments, yet whether modification of ecological niche traits collectively alters the pace of range extensions or contractions remains unknown. We quantified habitat niche, abundance, physiological performance and cellular defence/damage of range-extending coral reef fishes and coexisting local temperate fishes along a 2000 km latitudinal gradient. We also assessed their dietary and behavioural niches, and establishment potential, to understand whether ecological generalism facilitates successful range extension of coral reef fishes. The coral reef fish that increased all ecological niches, showed stronger establishment, increased physiological performance and cellular damage, but decreased cellular defence at their cold-range edge, whereas tropical species that showed unmodified ecological niches showed lower establishment. One temperate species showed decreased abundance, habitat niche width and body condition, but increased cellular defence, cellular damage and energy reserves at their warm-trailing range, while other temperate species showed contrasting responses. Therefore, ecological generalists might be more successful than ecological specialists during the initial stages of climate change, with increasing future warming strengthening this pattern by physiologically benefitting tropical generalists but disadvantaging temperate specialists.

## 1. Introduction

Ecological niche breadth is a key trait dictating a species’s adaptability to its environment and describes the range of environments or resources a species can use or inhabit [1]. Generalist species often have a wide niche breadth and use a broader suite of available resources compared to specialists who use a more restricted range of resources [2]. Ecological niche theory postulates that species abundance patterns reflect how well a particular environment meets the niche requirements of a species across multiple ecological axes; hence, species that can use a greater array of resources tend to be more widespread [3]. Therefore, specialist species are generally thought to be more vulnerable to extinction and rapid environmental change [4].

Species can respond to environmental disturbances in varying ways [5]. They can relocate to new environments [6], physiologically acclimate [7] and/or genetically adapt [8] to avoid the consequence of demographic collapse or extinction [9]. However, the balance between persistence and extinction is altered by climate change and has caused shifts in species range edges [10], impacting entire ecosystem functioning [11]. An essential component of an organisms' performance under global warming is their thermal tolerance, whereby organisms that experience higher temporal temperature fluctuations should have broader thermal tolerance ranges [12,13]. Hence, tropical, and polar species typically have narrower thermal tolerance ranges than temperate species [14,15]. Thermal tolerance range also varies within species based on age and life stage due to phenotypic plasticity [16]. Phenotypic plasticity includes behavioural, morphological and physiological modifications, which may enhance species performance in changing environments [16,17].

Although plasticity is observed as beneficial by optimizing performance under changing environments, it may not occur across multiple traits [18]. For example, behavioural plasticity such as changes in habitat use or altered species interactions could enable individuals to adjust to or avoid unfavourable environmental conditions, but a lack of physiological plasticity to broaden their thermal niche will be a disadvantage. Indeed, species with broader niches should respond quicker to changing environments and experience a faster rate of niche evolution [19], especially as species continue to track the pace of climate change.

Global warming is reshuffling marine and terrestrial species distributions worldwide [6,20]. In marine organisms, characteristics such as high propagule production and dispersal by ocean currents has led to faster range expansions than in terrestrial species under global warming [10,20,21]. Larval stages of marine organisms often track their thermal niches and colonize to suitable environments to avoid detrimental physiological effects of warming in their native habitats [22]. Regions associated with western boundary currents (e.g. East Australian Current in Australia, Kuroshio in Japan and Gulf Stream North-east America) are warming 2–3 times faster than the global average [23], and this has facilitated widespread tropicalization of high-latitude temperate ecosystems [24]. In Australia, the East Australian Current disperses coral reef fish larvae into temperate latitudes during warmer months (January to May) [25]. Winter temperatures largely prevent overwintering of these tropical fish due to thermal physiological constraints [26]. However, overwintering success is likely to increase as the East Australian Current continues to warm and intensify in strength [27,28].

The arrival of tropical species has disrupted temperate ecosystem functionality and stability [29,30]. Transition regions where tropical range-extending fish species overlap with local temperate communities has created novel species interactions [31–33], resource competition [34,35] and habitat modification [36]. Range-extending ecosystem engineers such as warm-adapted sea urchins and tropical herbivorous fish can cause community phase shifts where dominant habitat-forming kelp forests are overgrazed and eliminated, mediating a phase shift to barren-dominated ecosystems [30] and/or allowing the establishment of range shifting habitat-forming coral species and fishes [36,37]. Range extending and resident temperate species must, therefore, acclimate or adapt to changing and novel ecological conditions (i.e. habitat shifts, increased competition, and novel predators, prey and diseases) to persist [6]. During the initial phases of range extension, some tropical coral reef fishes are known to modify their behavioural [38] or dietary [39,40] niche breadths, ultimately reducing overlap with local temperate fish species and filling separate niche space [41]. Past studies often considered individual traits of ecological niches; however, to accurately understand which tropical species are most successful range extenders and which temperate species may resist tropical species disturbances, multiple ecological niche traits should be studied collectively.

Here, we investigate whether ecological generalism and physiological responses may benefit tropical coral reef fish range extensions into novel temperate ecosystems. Our findings integrate previous analyses focusing on individual niche traits [38–40] that found range-extending coral reef fishes either adjust their behavioural or dietary niches in novel temperate environments. It remains unclear whether niche modification of these range-extending species occurs along a single niche axis or across multiple niche traits as they expand into novel temperate ecosystems. We assessed multiple niche traits *in situ* at a global warming hotspot in eastern Australia for four range-extending coral reef fish species and three sympatric temperate fish species along a 2000 km latitudinal gradient from tropical, sub-tropical, warm temperate and cold temperate environments. We quantified habitat niche breadth from field observations and combined this with data from our previous niche studies (behaviour and diet) focusing on the same species and study sites to understand whether (i) successful tropical range extenders are more likely to be ecological generalists, (ii) local temperate fish species can adjust multiple ecological niches at their warm-trailing edge in response to ocean warming and range-extending competitors, and (ii) the physiological and cellular performance of both tropical and temperate species is compromised towards their range edges. Understanding multi-species ecological niches and physiological responses to environmental change is critical to predicting future community structures under climate change.

## 2. Methods

### (a) Study species

**Figure 1.**
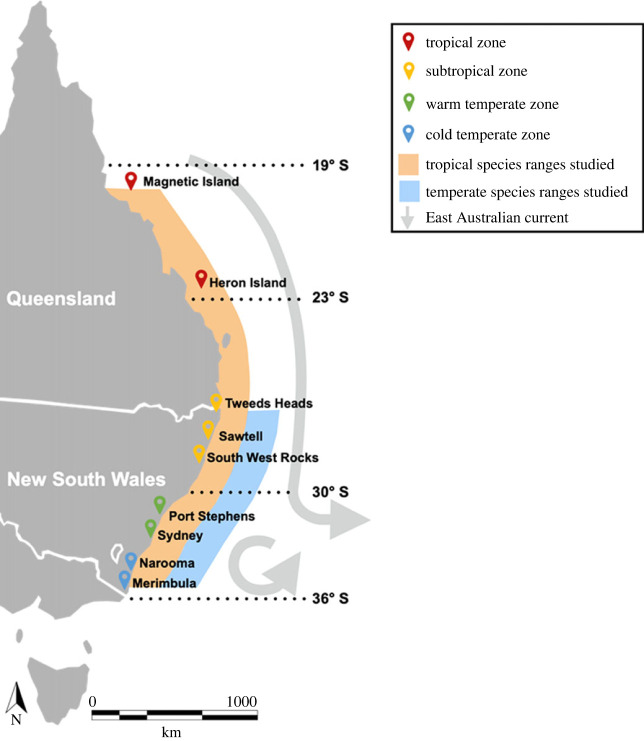
Map of the study sites along the east Australian coastline. Grey arrow indicates the direction of the East Australian Current that disperses tropical larvae from the Great Barrier Reef and Solitary Islands into temperate ecosystems. Red location markers indicate sites in the tropical region: Magnetic Island (19.1° S) and Heron Island (23.4° S). Orange location markers indicate sites in the sub-tropical region: Tweeds Heads (28.2° S), Sawtell (physiological analysis only, 30.4° S) and South West Rocks (30.9° S). Green location markers indicate sites in the warm temperate region: Port Stephens (32.7° S) and Sydney (33.8° S). Blue location markers indicate sites in the cold temperate latitude region: Narooma (36.2° S) and Merimbula (36.9° S).

We selected the most prevalent and abundant range shifting coral reef fish species for this study [40]: two omnivorous fishes, the sergeant major damselfish (*Abudefduf vaigienis*), scissortail sergeant (*Abudefduf sexfasciatus*) and two herbivores, the dusky surgeonfish (*Acanthurus nigrofuscus*) and convict tang (*Acanthurus triostegus*). These tropical coral reef fish have been observed in temperate and sub-tropical environments forming mixed-species shoals with morphologically similar temperate fish species [31]. We chose three of the most common local temperate species, the Australian mado (*Atypichthys strigatus*), stripey (*Microcanthus strigatus*) and white-ear scalyfin (*Parma microlepsis*), the first two of which school with the *Abudefduf* species. These seven target species of tropical and temperate fish species have coexisted seasonally (summer and autumn months) in temperate regions over at least the past approximately 20 years (figure 1) [25], but coexist throughout the entire year in sub-tropical regions [38].

### (b) Study locations

Nine study sites in four range zones were selected along a 2000 km latitudinal gradient across the east Australian coastline during April–July 2021 to encapsulate the leading range edge of the tropical and trailing range edge of the temperate fish species (figure 1). The tropical zone sites (latitude range: 19.1–23.4° S) reflects the coral reef species native range and the absence of the temperate fish species. The sub-tropical zone sites (latitude range: 28.2–30.9° S) reflect the tropical species’ most southern region where breeding adults occur, and the temperate fish species warm-trailing range edge, where they coexist during all seasons with the coral reef fishes [27,38]. The warm temperate zone sites (latitudinal range: 32.7–33.8° S) is considered a tropicalization hotspot where coral reef fish presence has increased for at least two decades [25] and where tropical and temperate fish species coexist seasonally (summer and autumn). The cold temperate zone (latitudinal range: greater than 36.9° S) is the most southern and novel temperate environment for coral reef fishes (cold-range edge), where temperate fish density is higher than tropical fish density and where most tropical fishes fail to overwinter [25,35]. Available habitats at each location were defined following Coni *et al*. [36]: kelp forests (dominated by *Ecklonia radiata*), turf-forming algae (less than 10 cm in height), oyster reefs (dominated by *Saccostrea glomerata*), barrens (coralline algae encrusted rock shelf, artefact of overgrazing by invading tropical herbivorous fish species and sea urchins *Centrostephanus rodgersii*) and coral reefs.

### (c) Fish collection

One model species from each temperature affinity (one tropical and one temperate species) were selected for the physiological analyses. The tropical species *Abudefduf vaigiensis* was the most abundant range-extending coral reef fish [42] which commonly co-shoals with morphologically similar temperate species *A. strigatus* [31]. These species are core model species used by previous studies [32,33,43]. Fishes were collected using 9 : 1 ethanol : clove oil spray, hand and seine nets during April–July 2021. Collected fishes were held in a 50 l bucket filled with fresh seawater and an aerator. Fishes were then euthanized using the *iki jime* method and wet weight (±0.01 g) and standard length (±0.01 mm) were recorded (see electronic supplementary material, table S1 for replicates). Fishes were kept frozen at −20°C during field collection (April–July 2021) and then stored at −80°C until further processing. While the preferred long-term storage for enzyme measures is −80°C, previous research has demonstrated these measures can remain stable during short-term storage at −20°C [44,45]. Therefore, since all samples were stored at these conditions consistently, the short-term storage at −20°C should not impair the interpretation of relative change of cellular defence and damage along the latitudinal gradient.

### (d) Fish abundance and habitat niche

Visual surveys were used to estimate the abundance of tropical coral reef fish and temperate fish across patches of available habitats (see electronic supplementary material, table S2 for replicates). Snorkelers swam along a 10 m (length) belt transect, counting the abundance of the seven target fish species and visually estimated the individual fish size classes of less than 5, 5–10, 10–20, 20–30, greater than 30 cm within 2 m (width) on each side (40 m^2^ area per transect). Mobile target fish species were first counted, and their size (total length) estimated, followed by an extensive search for smaller-bodied target fish hiding within rocks, crevices and kelp leaves. All visual surveys were performed during the morning and afternoons at depths of 0.5–3 m. The sample size of visual surveys conducted across habitat types is representative of the habitat availability at each location. For example, a sample size of five transects (40 m^2^ per transect) indicates there is only 200 m^2^ of habitat available at the location. Relative habitat use of each species across the latitudinal range was determined by calculating abundance in each habitat type and expressed as a percentage by dividing the abundance in each habitat by the total abundance of the species observed at the site (i.e. across all habitats).

Shannon Wiener index of diversity W=Σ(−logP∗P) was calculated as an index of habitat niche width, where *P* is the proportional habitat use of each species within all available habitats at the site. This method accounts for the total number of available habitats used by a species at each site, and the frequency (abundance) at which species uses the available habitats. The fewer habitats a species uses at a site relative to the total number of available habitats the lower the index value (narrow niche width, i.e. habitat specialist) and the more habitats a species uses, the higher the index value (wide niche width, i.e. habitat generalist). The number of habitat types at each site does not influence the index of the values.

## 3. Physiological proxies

### (a) Cellular defence and damage

Muscle tissue (approx. 70 mg) was used for a 10% tissue homogenate (1 : 9 ratio of muscle tissue to phosphate-buffered saline solution) to assess total protein (TP) content, total antioxidant capacity (TAC) and malondialdehyde concentration (MDA). TAC is an indicator of cellular defence and MDA is an indicator of cellular damage [46], collectively low TAC and high MDA indicate high oxidative damage. Levels of cellular defence and damage are influenced by environmental factors such as water temperature, oxygen availability and salinity [47], therefore, variability in cellular defence and damage can reflect differences among individual fishes on their response to environmental change along a latitudinal gradient. TP was calculated through the Coomassie brilliant blue method and measured with Jenway 6405 spectrophotometer at absorbance (optical density, OD) 595 nm. Total protein concentration was then used to calculate TAC (OD 520 nm) and MDA (OD 532 nm) following manufacturers protocols. Elabscience (China) assay kits were used to calculate TP (catalogue number: E-BC-K168-S), TAC (catalogue number: E-BC-K136-S) and MDA (catalogue number: E-BC-K025-S) and were calculated as follows:

$$\mathrm{TP}(g\;\;l^{-1})=\;\frac{\mathrm{ODsample}-\mathrm{ODblank}}{\mathrm{ODstandard}-\mathrm{ODblank}}\times \mathrm{standard}\;\mathrm{conc}.\;(0.563\;g\;\;l^{-1}),$$

$$\begin{array}{rl} \mathrm{TAC}\;(U\;\mathrm{mg}{\;}^{-1}\;\mathrm{protein})\; & =\;\frac{\mathrm{ODsample}\;-\;\mathrm{ODcontrast}}{0.01}\\ & \;\;÷\;30\;\times \;\;\mathrm{volume}\;\mathrm{of}\;\mathrm{homogenate}\\ & \;\;÷\;\mathrm{protein}\;\mathrm{conc}.\;\left(\mathrm{mg}\;\mathrm{protein}\;ml^{-1}\right), \end{array}$$

$$\begin{array}{ccc} \mathrm{MDA}\;(\mathrm{nmol}\;mg^{-1}\;\mathrm{protein})\; & =\; & \frac{\mathrm{ODsample}-\mathrm{ODcontrast}}{\mathrm{ODstandard}-\mathrm{ODblank}}\;\\ \; & \; & \times \;\mathrm{standard}\;\mathrm{conc}.\;(10\;\mathrm{nmol}\;ml^{-1})\;\\ \; & \; & ÷\mathrm{protein}\;\mathrm{conc}.\;(\mathrm{mg}\;\mathrm{protein}\;ml^{-1})\; \end{array}$$

### (b) Fulton's condition index

Wet weight (WW ± 0.01 g) and standard length (SL ± 0.01 mm) were measured after the fishes were euthanized. Fulton's condition index was calculated to assess the body condition, and was calculated as follows:

$$\mathrm{Fulto}n^{\prime }s\;\mathrm{condition}\;\mathrm{index}=\frac{\mathrm{WW}\;(g)}{\mathrm{SL}\;{\left(\mathrm{mm}\right)}^{3}}\times 100.$$

### (c) Hepatosomatic index

Livers were dissected from frozen fishes, fixed in 100% ethanol for approximately 24 h then removed and weighed to the nearest 0.0001 g. Hepatosomatic index was calculated as the ratio between liver weight and body weight, and expressed as a percentage. Hepatosomatic index is an indicator of short-term energy storage [48] and calculated as follows:

$$\mathrm{Hepatosomatic}\;\mathrm{index}=\frac{\mathrm{weight}\;\mathrm{of}\;\mathrm{liver}\;(g)}{\mathrm{wet}\;\mathrm{weight}\;\mathrm{of}\;\mathrm{fish}\;(g)}\times 100.$$

### (d) Statistical analyses

Linear regression models were used to quantify changes in habitat niche and abundance along a latitudinal gradient. We then used linear models with a quadratic term to assess changes in physiology along the latitudinal gradient. We first considered a model combining wet weight of the fish (to disentangle potential body-size effects) and the quadratic term of latitude (to account for possible nonlinear, bell-shaped response curves characteristic of biological optima). Combinations of covariates were compared based on the Akaike information criterion (AIC) and top-ranked models of each physiological proxy were retained for the final models (electronic supplementary material, table S3). Linear regression models were performed on log-transformed data to reduce the influence of a few high values and tested separately for each species. Assumptions of constant variance and normal residual distribution were validated based on diagnostic plots including residual deviations against fitted values and normal QQ plots. Differences in relative fish abundance across available habitats (turf, barren, oyster, kelp and coral) for the tropical and temperate species at the different regions (tropical, sub-tropical, warm temperate and cold temperate) were assessed using non-metric multidimensional scaling (nMDS) based on Bray–Curtis dissimilarly and 9999 permutations. We removed one datapoint (*A. sexfasciatus* at latitude 19.1° S) from the nMDS analysis since it was the only species that occupied 100% coral and skewed the stress of the nMDS. We used R (v.4.3.1) [49] and the ‘lm’ function [50] for linear regression model analysis, ‘vegan’ functions for nMDS and ‘ggplot2’ package for graphical outputs.

## 4. Results

### (a) Niche modifications and habitat association of tropical fishes at their cold-range edges

**Figure 2.**
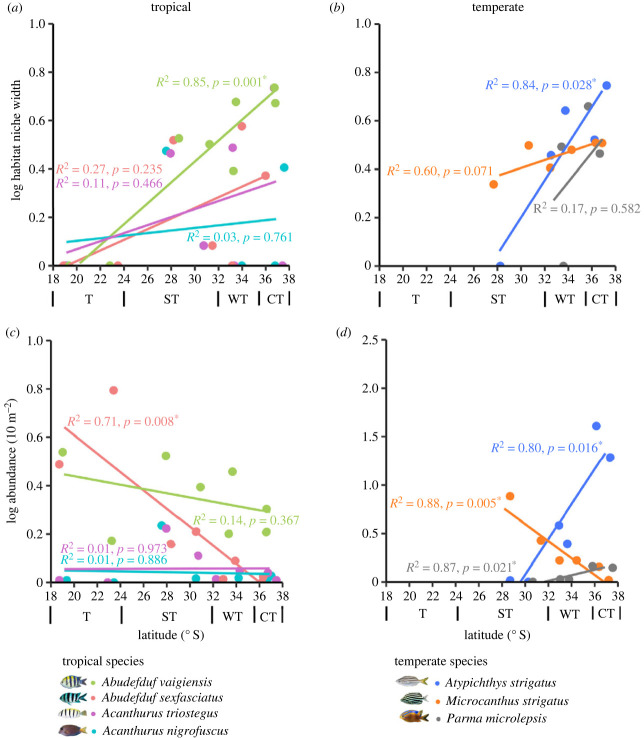
Habitat niche width (Shannon–Weiner index) and average abundance (10 m^−2^) across a latitudinal gradient for (*a*,*c*) tropical and (*b*,*d*) temperate fish species. ‘T’ represents the tropical region (latitudes 19.1–23.4° S), ‘S’ represents the sub-tropical region (latitudes 28.17–30.89), ‘WT’ represents the warm temperate region (latitudes 32.76–33.86) and ‘CT’ represents the cold temperate region (latitudes 33.8–36.8° S). Fitted regression lines were calculated using log-transformed data and significance represented by * (*p* < 0.05). Points on the *x*-axis have been jittered. See electronic supplementary material, figures S4 and S5 for 95% confidence intervals, and electronic supplementary material, tables S4 and S5 for regression outputs.

*Abudefduf vaigiensis* was the only fish out of the four tropical species that increased its habitat niche width towards their cold-range edge (figure 2*a*; *p* = 0.001, *R*^2^ = 0.85; electronic supplementary material, table S4), while the other three species (*A. sexfasciatus*, *A. nigrofuscus* and *A. triostegus*) showed no significant change in habitat niche width with increasing latitude. The abundance of *A. sexfasciatus* decreased with latitude (figure 2*c*; *p* = 0.008; *R*^2^ = 0.71, electronic supplementary material, table S5), but that of the other species did not change.

Turf, barren and oyster habitats appeared to be the main habitats occupied across the tropical fish species latitudinal range (electronic supplementary material, figure S1). At the cold temperate zones, all tropical species were mostly associated with oyster habitats (electronic supplementary material, figures S1 and S2). At the warm temperate zone, *A. vaigiensis*, *A. sexfasciatus* and *A. nigrofuscus* were mostly associated with barren habitats, while *A. triostegus* was associated with turf and oyster habitats. At the sub-tropical zone, barren and turf habitats were mostly occupied by *A. vaigiensis*, *A. sexfasciatus* and *A. nigrofuscus* and turf and oyster were occupied by *A. triostegus*. At their tropical range, *A. vaigiensis* associated the most with barren, *A. sexfasciatus* with barren and coral and *A. nigrofuscus* and *A. triostegus* with turf habitats.

### (b) Niche modifications and habitat association of temperate fishes at their warm-trailing edges

*Atypichthys strigatus* showed a decreased habitat niche width (figure 2*b*, *p* = 0.028, *R*^2^ = 0.84, electronic supplementary material, table S4) and abundance (figure 2*d*; *p* = 0.016, *R*^2^ = 0.80; electronic supplementary material, table S5) with decreasing latitude towards their warm-trailing edge. The other two temperate fish species *M. strigatus* and *P. microlepsis* showed no change in habitat niche width as a function of latitude, but experienced increased (*p* = 0.005, *R*^2^ = 0.88; electronic supplementary material, table S5) and decreased (*p* = 0.021, *R*^2^ = 0.87; electronic supplementary material, table S5) abundance, respectively, towards their warm-trailing edges.

The temperate species were not associated with a single habitat type across their latitudinal range (electronic supplementary material, figures S1 and S2). At their warm-trailing edge, *A. strigatus* was mostly associated with turf habitats, *M. strigatus* with turf and oyster and *P. microlepsis* was not observed at these latitudes. At the warm temperate region, *A. strigatus* was observed the most in kelp habitats, and *M. strigatus* and *P. microlepsis* with barren and turf habitats. At the cold temperate region, *A. strigatus* associated the most with barren and kelp habitats, *M. strigatus* with barren and oyster and *P. microlepsis* with barren, kelp and turf habitats.

### (c) Physiological and cellular responses of tropical and temperate fishes at their range edges

**Figure 3.**
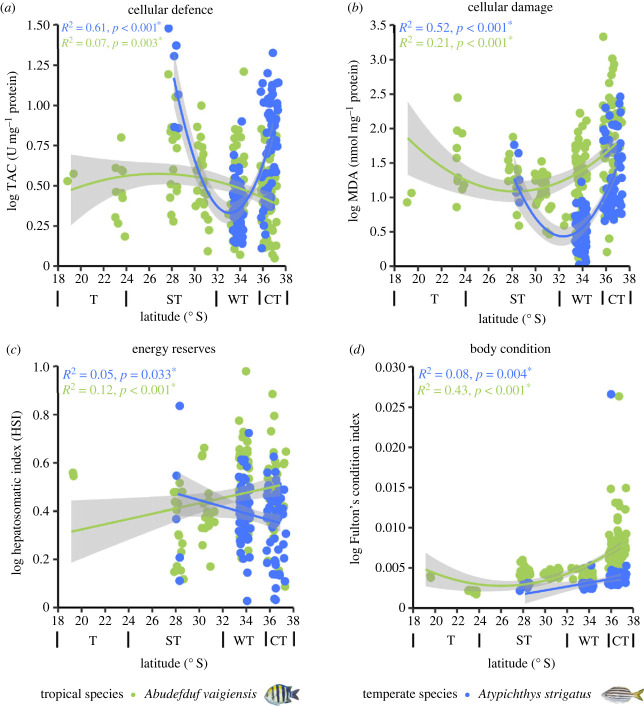
(*a*) Cellular defence (TAC), (*b*) cellular damage (MDA), (*c*) energy reserves (hepatosomatic index) and (*d*) body condition (Fulton's condition index) across a latitudinal gradient for tropical (*A. vaigiensis*) and temperate (*A. strigatus*) fish species. Regression lines (quadratic or linear) were fitted based on the top model according to AIC (see electronic supplementary material, table S3) and calculated using log-transformed data. An asterisk denotes significant relationships (*p* < 0.05). Points on the *x*-axis have been jittered. See electronic supplementary material, table S6 for regression outputs.

Tropical species *A. vaigiensis* showed decreased cellular defence (TAC, figure 3*a*; *p* = 0.003, *R*^2^ = 0.07; electronic supplementary material, table S6), and increased cellular damage (MDA, figure 3*b*; *p* = <0.001, *R*^2^ = 0.21), energy reserves (HSI, figure 3*c*; *p* < 0.001, *R*^2^ = 0.12) and body condition (Fulton's condition index, figure 3*d*; *p* < 0.001, *R*^2^ = 0.43) towards their cold-range edge. Temperate species *A. strigatus* showed increased cellular defence (TAC, figure 3*a*; *p* = <0.001, *R*^2^ = 0.61; electronic supplementary material, table S6), oxidative damage (MDA, figure 3*b*; *p* < 0.001, *R*^2^ = 0.52) and energy reserves HSI, figure 3*c*; *p* = 0.033, *R*^2^ = 0.05) and decreased body condition (Fulton's condition index, figure 3*d*; *p* = 0.004, *R*^2^ = 0.08) towards their warm-trailing range edge. Both tropical (electronic supplementary material, figure S3 and table S6; *p* < 0.001, *R*^2^ = 0.12) and temperate (electronic supplementary material, figure S3 and table S6; *p* < 0.001, *R*^2^ = 0.32) species showed decreased wet weight towards their leading and trailing range edges, respectively.

## 5. Discussion

Habitat niche modification may alter future populations in tropicalization hotspots where novel community compositions are emerging from intermixing of tropical range-extending species with temperate species at their trailing edges. One of the most common range-extending coral reef fish species (*A. vaigiensis*) showed an increased habitat niche width towards its cold-range edge, while one co-schooling temperate species (*A. strigatus*) experienced decreased habitat niche breadth as well as abundance towards its warm-range edge. Habitat niche expansion reflects increased generalism, whereby habitat generalists may be more capable to invade novel habitats and maintain their populations than habitat specialists that may have restricted capacity to cope with habitat shifts under future climate change [52] (electronic supplementary material, figure S5). This could suggest that this tropical species may have better capacity to exploit different, as well as novel habitat types, and share niche space with the temperate species at higher latitudes [41]. By contrast, temperate fish populations that suffer from decreased habitat niches may experience abundance declines at their warm-trailing range edge under habitat shifts due to ocean warming, as observed here for *A. strigatus*. Therefore, species with a broader capacity to modify their habitat niche towards their range edges may better adapt to novel and/or changing ecosystems under ocean warming and in climate mixing zones.

Reshuffling of species abundance patterns may reflect future community structure under climate change. Here, three of the four tropical species showed no abundance change, while one showed decreased abundance towards their cold-range edge, and two of the three temperate species showed decreased abundance, while one temperate species showed increased abundance towards their warm-trailing range edge. Since range edge populations often experience more extreme environmental conditions (i.e. water temperatures and changing habitats) than their central populations, lower abundances are expected at range edges when maximum plasticity to the environment is reached [53]. This suggests that some tropical and temperate species may either have low plasticity potential towards their cold- or warm-trailing range edges, or that their plasticity limits have been reached. While present-day fish abundance patterns may be useful in detecting future population changes at range edges, the ability to express ecological generalism in changing environments may override these patterns and strengthen species persistence under future climate change.

**Figure 4.**
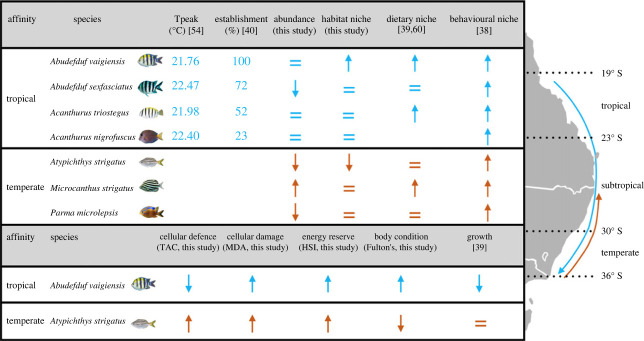
Conceptual diagram showing significant patterns (*p* < 0.05) for establishment (see electronic supplementary material, table S7 for calculation, values represent the per cent of their establishment at their cold-range edge), abundance, the three ecological niches: habitat, dietary and behavioural, respectively, and the cellular and physiological metrics (cellular defence, cellular damage, energy reserves, body condition and growth). The symbols show the significant increase (↑), decrease (↓) or no change (=) for the tropical species towards their cold-leading range edge (blue) and the temperate species towards their warm-trailing range edge (orange), respectively. The tropical species range studied here is shown by the blue arrow encapsulating their home range (tropical and sub-tropical) and their cold-leading range edge (temperate), and the temperate species range is shown by the orange arrow encapsulating their home range (temperate) and their warm-trailing range edge (sub-tropical). *T*_peak_ is the temperature at which abundance of this species declined, the lower the value, the more cold-tolerant the species is.

Modification of multiple ecological niche traits might be more beneficial than modification of single ecological niche traits in novel ecosystems. A prevalent range-extending tropical fish species (*A. vaigiensis*) broadened three ecological niches (habitat, dietary and behavioural) towards its cold-range edge in contrast to the other studied tropical species [38–40]. Indeed, ecological generalism may enhance successful establishment potential of this species in novel temperate ecosystems (figure 4) since it is also more abundant (this study), established [40], observed overwintering [54] and more cold-tolerant [54] at its cold-range edge than the other range-extending tropical species. By contrast, three other tropical species either broadened two ecological niches (dietary and behavioural: *A. triostegus*) or just one niche (behavioural: *A. sexfasciatus* and *A. nigrofuscus*) and showed lower establishment and cold-tolerance than *A. vaigiensis* at their cold-range edge (figure 4). None of the three temperate species altered all three niches together towards their warm-trailing range edges (figure 4). Temperate fish species *M. strigatus* and *A. strigatus* modified two ecological niche traits towards their warm-trailing range edge; however, *A. strigatus* decreased its habitat niche width. One temperate species (*P. microlepsis*) increased one niche trait (behavioural) and is known to shift to deeper habitats towards its warm-trailing range edge [55]. Previous studies have shown that habitat requirements are a core determinant of tropicalization [41,52]. Here we cannot distinguish whether habitat niche modification alone or alterations of multiple niches determines persistence at range edges in changing ecosystems. The species that showed the strongest positive (tropical *A. vaigiensis*: increased establishment) and negative (temperate *A. strigatus*: decreased abundance) responses, were also the only species that showed increased and decreased habitat niche width at their cold-range and warm-trailing range edges, respectively. Nevertheless, species that have the ability to modify multiple ecological niche traits are likely to enhance their success under climate change, as the ecological impacts of warming expand beyond that of just habitat regime shifts.

Reduced ability to shift habitat utilization may limit species persistence at novel ranges experiencing climate-driven habitat phase shifts, although specialist species that already occupy future prevailing habitat types may be advantaged under future climate change. Here, all tropical and temperate fish species were observed inhabiting the future projected turf-dominated habitat [36] to some degree across their ranges, suggesting that these species may better placed in adjusting to future habitat shifts under climate change than for example kelp or coral specialists. Ocean warming has already directly (marine heatwaves) and indirectly (overgrazing by range shifting sea urchins and herbivorous tropical fish species) facilitated the loss of kelp forests [30,56], whereby range-extending tropical fish species will likely benefit more than temperate fish species from the phase shift of kelp-dominated temperate habitats to barren-dominated habitats [36]. Yet, ocean acidification may buffer such phase shifts, by directly inhibiting urchin abundance [36] and indirectly boost algal productivity [57], ultimately facilitating another phase shift towards simplified turf-dominated ecosystems [36,58,59]. Turf-dominated ecosystems are favoured by temperate fish species more than tropical fish species (besides herbivorous fish species), which may slow the pace of tropical range shifts and benefit that of temperate fishes [36]. Nevertheless, species which currently have the capacity to occupy turf habitats may still benefit or sustain their populations under future habitat phase shifts.

Niche expansions and plasticity can facilitate successful range extensions, yet the concurrent effects of climatic stress on physiological function may slow the ability of species persistence in novel ecosystems. The most prevalent range-extending coral reef fish (*A. vaigiensis*) showed increased cellular damage, body condition and energy reserves and decreased cellular defence towards their cold-range edge. For the other three tropical species, previous studies found no changes in body condition and decreased performance (feeding and growth) towards their cold-range edge compared to their core range [32,43,60]. The tropical species (*A. vaigiensis*, *A. sexfasciatus* and *A. triostegus*) also reduced their activity levels and feeding towards their cold-range edge, which may suggest a behavioural strategy to preserve and maintain body condition and energy reserves [32]. However, increased cellular damage may dampen this strategy by diverging energy away from important fitness related traits (e.g. growth, reproduction and survival; [47,61,62]), which may slow the current establishment of these species in novel temperate ecosystems. Yet, future summer temperatures may alleviate this reduced physiological functioning when water temperatures track their optimum thermal range [63]. However, future winter temperatures may still have limiting effects on physiological, behavioural and cellular function of range-extending tropical fishes at their cold-range edge, which may seasonally limit and slow the persistence of these tropical species under future climate change [63,64]. In contrast to tropical species, the most common shoal-mate of *A. vaigiensis* (i.e. the temperate *A. strigatus*) showed increased cellular defence, cellular damage, energy reserves and decreased body condition towards their warm-trailing range edge (figure 4). The observed increased cellular defence may counteract the increased cellular damage and alleviate physiological constraints, although their abundance declines at their warm-trailing range edge may continue to limit their persistence. The other temperate species showed no changes in body condition or performance towards their warm-trailing range edge (besides *M. strigatus*, which showed higher growth [32,43]). Reduced physiological function and abundance declines may currently limit the performance of some temperate fishes and result in range contraction at their warm-trailing range edge, although these temperate fishes may have higher advantages than tropical species during future winter temperatures [64]. While both tropical and temperate fish species experience direct physiological responses to ocean warming, ecological generalism may be a stronger mediator of their abundances under climate change.

## 6. Conclusion

We show that the most prevalent range-extending coral reef fish species present in temperate south east Australian ecosystems exhibited plasticity across multiple niches but suffered from increased cellular damage and decreased cellular defence at their cold-range edge. Hence, we conclude that ecological generalism could be an important trait for invading novel climate environments, even under conditions that create physiological stress. By contrast, sympatric temperate species showed a lower degree of ecological generalism while also showing increased cellular damage and cellular defence at their warm-trailing edge. Such contrasting patterns in phenotypic plasticity and physiological functioning could be strong mediators of population changes and species interactions in rapidly warming temperate ecosystems.

## Data Availability

The data that support the findings of this study are provided in electronic supplementary material [51].
